# When theory meets reality- a mismatch in communication: a qualitative study of clinical transition from communication skills training to the surgical ward

**DOI:** 10.1186/s12909-023-04633-2

**Published:** 2023-10-04

**Authors:** Leif Berglund, Johanna von Knorring, Aleksandra McGrath

**Affiliations:** 1 Department of Clinical Sciences, Umeå- University, Umeå, Sweden; 2 Department of Surgical and Perioperative Sciences, Umeå University, Umeå, Sweden

**Keywords:** Medical education, Knowledge transfer, Communication skills, Patient centered care, Qualitative study, Sweden

## Abstract

**Background:**

Communication skills training in patient centered communication is an integral part of the medical undergraduate education and has been shown to improve various components of communication. While the effects of different educational interventions have been investigated, little is known about the transfer from theoretical settings to clinical practice in the context of communication skills courses not integrated in the clinical curriculum. Most studies focus on single factors affecting transfer without considering the comprehensive perspective of the students themselves. The aim of this study is to explore how the students experience the transition to clinical practice and what they perceive as challenges in using patient centered communication.

**Methods:**

Fifteen 4th year medical students were interviewed 3 weeks after the transition from an advanced communication skills course to surgical internship using semi-structured interviews. Qualitative content analysis was used to analyze the interviews.

**Results:**

The analysis resulted in a theme ‘When theory meets reality- a mismatch in communication’. It was comprised of four categories that encompassed the transfer process, from theoretical education, practical communication training and surgical internship to students’ wishes and perceived needs.

**Conclusions:**

We concluded that preparing the students through theoretical and practical training should reflect the reality they will face when entering clinical practice. When educating medical students as a group, their proclivity for perfectionism, high performance environment and achievement-related stress should be taken into consideration. The role of tutors being role models, offering guidance, giving feedback and providing support plays a major part in facilitating transfer of communication skills. To enable transfer to a larger extent, the environment needs to promote patient centeredness and students need more opportunities to practice communication with their patients.

## Introduction

Delivering communication skills education to medical students is important as it responds to a shift in the approach towards patients in healthcare, from a biomedical view to a broader biopsychosocial viewpoint [1]. Research findings indicate that the communication between physician and patient affects health related outcomes, such as treatment outcomes, compliance with treatment, patient empowerment and patient satisfaction [2, 3]. While the literature supports the importance of effective communication between physician and patient, and education in communication skills has become an integral part of the curriculum in undergraduate medical education, there is no consensus on how the communication skills are best taught or evaluated [4] Despite a trend for the communication skills teaching to gradually move toward experiential and integrated approaches [4], communication skills are often taught as separate courses, not integrated in the clinical curriculum, and delivered by faculty with special interest in communication skills.

With non-integrated communication skills courses the potential issue is transfer of communication skills to clinical practice. In such settings, transfer occurs between two environments, one affected predominantly by the formal curriculum, the other with strong influence of hidden curriculum. When moving between theoretical and practical setting, we assume that learning is influenced strongly by the concept of communities of practice, professional learning and situated learning [5]. Learning becomes a social process and a situated activity with medical students participating in communities of practice and moving toward integration in their sociocultural practices.

While transfer of communications skills in postgraduate training was shown to be dependent on a number of factors [6], some of which are; trainee’s perception of positive outcomes, clinical supervision during initial application of skills [7], amount of time for tutoring and attitudes of senior colleagues [8] and frequent changes in clinical learning environments [9] [16], there is a lack of studies exploring specifically the undergraduate context. Mapped against Kirkpatrick’s model for measuring the effectiveness of interventions [10], most studies examine only whether learners acquire the intended knowledge and skills at the end of educational intervention, with a lack of studies investigating longer term behavioral changes, influence on the organization and benefits for stakeholders [6]. What is also not known at medium term, is how do medical students try to incorporate newly acquired communication skills into their interactions with patients in a clinical setting and how to facilitate this process.

This qualitative interview study focuses on exploring how the medical students experience the transition from a non-integrated communication skills course to the clinical practice. The goal of this study is to bring to light factors which students themselves perceive as beneficial or disadvantageous. Furthermore, this study aims to identify areas of potential improvement in the design of curriculum which could enhance retention and application of communication skills.

## Materials and methods

### Theoretical framework

A qualitative approach was chosen as it is well suited to conducting exploratory work into the phenomenon where not much is known [11]. As all of the researchers have a background in medical education, either as providers (tutor and faculty member) or consumers (medical student), our methodological choices required an approach which would enable us to explore the phenomenon of transfer of skills from the perspective of medical students experiencing the process, while acknowledging our pre-understanding, allowing us to bring it forward and use it positively in all stages of the research process, from research question to data collection, analysis and writing. After also considering the degree of transformation of data, we have settled on qualitative method analysis [12], with relatively low level of data interpretation to capture the nuances in experiences of participants [11].

### Setting

The study was conducted in northern Sweden, at the University Hospital of Umeå. The study participants were 4th year medical students from the Medical Program at Umeå University. The undergraduate medical education is five and a half years long and consists of five pre-clinical, five clinical and one research semester. Students rotate through internships at different departments at the regional hospitals and primary care centres. The communication skills teaching is organized as part of the professional development course (PD), which spans the entire undergraduate medical program, and focuses on communication- and leadership skills, self-reflection, ethics, gender studies and discrimination in healthcare. The course is both theoretical and practical and involves lectures, reflective writing and exercises in communication skills with roleplaying. The communications skills courses start with introduction to patient centred communication (PCC) during pre-clinical part of the program and are not integrated with the clinical internships. The communication skills are taught by tutors with special interest in communication skills rather than regular faculty.

PCC is a theoretical base used not only by Umeå university but also by other Swedish universities for shaping communication skills teaching. Specifically, Larsen’s five cards patient centred communication model (PCCM), which emphasizes patient perspective and structures the consultation into three parts (patient’s, physician’s and shared part) is used [13]. In PCC the focus is on eliciting the patient’s perspective, emphasizing the patient’s autonomy and involving the patient in his/her own care. After the recent national overhaul of medical curriculum, assessment of PCC skills is included in obligatory Entrustable Professional Activities, which are to be examined broadly by all clinical tutors [14]. At present, PCC skills vary between tutors from different clinical specialities, with primary care having highest number of physicians trained in the PCCM thanks to having introduced PCC education as part of residency program.

The communication skills course directly preceding the surgical internship taught during the 4th year focuses on application of the PCCM and advanced communication skills, such as breaking bad news, through peer roleplay, self-reflection and feedback. It consists of two days of exercises in groups of nine students, who together with two communication skills tutors provide feedback after roleplaying.

Throughout the consultation the physician is expected to validate the patient and their experience using receipts (phrases signaling attentive listening and acceptance) and summaries (patient’s own words showing that the physician has understood the patient).

### Recruitment

Only those medical students who participated in the advanced communication skills course directly prior to the start of surgical internship were eligible for participation in the study.

The students were recruited via email to all 4th year students as well as personal emails to those who were later identified as eligible i.e., placed directly after the communication skills course in the surgical ward. Following that, three medical students were encouraged to contact the researchers by other participants, who were interviewed for the study.

There were 10 female and 5 male participants in this study. This distribution is close to gender distribution of Swedish medical graduates in recent years (57% women and 43% men). Average age of the participants was 26,4 years. The interviews were performed after three weeks of the internship on the surgical ward.

### Data collection and analysis

Data collection was done through one-on-one, in depth, semi-structured interviews performed through video link and only the audio from interviews was recorded (see Table 1 for interview questions). Each interview took between 30 min to an hour. The number of interviews was not pre-determined, rather they were conducted until data saturation was achieved. After initial 2 interviews, the data was analyzed, and the interview questions were finetuned to better focus on factors relevant to clinical transition in subsequent interviews.

The research team consisted of three researchers; Leif Berglund who at the time was a medical student (LB), Aleksandra McGrath, MD, a communication skills teacher and senior researcher (AM) and Johanna von Knorring, MD, a doctoral student with focus on qualitative methods and tutor in communication skills (JvK). Interviews were conducted by LB. JvK, and AM contributed to the analysis as described below.

Analysis of the data was done with qualitative content analysis method according to Graneheim and Lundman [12, 15]. The interviews were transcribed verbatim in Swedish. The transcripts were initially read through several times independently by the researchers, to get an overarching sense of the data. Meaning units were identified individually and then coded separately by LB, AM and JvK.

After initial coding, the research group compared codes and memos and discussed their overall understanding of the data. The codes were then scrutinized and divided into preliminary subcategories until the research group reached consensus. The research group met continuously throughout the analysis process. Categories were subsequently constructed according to the subcategories, making sure that one subcategory could not fit into several categories. During the process the research group worked with constant comparison of codes, subcategories, and categories, aiming for consensus while considering diverse interpretations. When the categories were finalized, a theme connecting all four categories was formed (see Table 1). After data analysis two participants were asked whether the results were representative of their narratives. No changes to the text were suggested after member checking.

**Table 1:** Questions included in the semi-structured interview guide

Interview guide	
1. In your opinion, what is the message the professional development course is trying to convey? 2. What is your view on communication skills teaching and the professional development course? 3. How would you describe your experience regarding applying what you have learned during communication skills course during your time on surgical ward? 4. Have you been able to interact with patients and use the patient centered communication model during your internship at the surgical ward? 5. Have there been any obstacles for you to use the patient centered consultation model? 6. What do you think would ease the transition from educational setting to clinical setting? 7. What would you say is missing from your education, regarding communication skills teaching?	

## Results

After the data was analysed, an overarching theme ***When theory meets reality, a mismatch in communication*** was constructed. This theme consists of four categories; *Conceptual understanding and attitudes towards PD, Experiences from theoretical communication training, Reality of clinical transition* and *Wishes and perceived needs* (see Table 2). The resulting theme describes factors both intrinsic and extrinsic in nature, affecting clinical transition of communication skills. The factors varied from preconceptual understandings about communication skills training and the PD course, opinions and experiences regarding communication skills training, challenges with application of the PCCM and impact of mentors to expressed wishes regarding changes needed in PD education and surgical internship. We will present the four categories with their respective subcategories in italics, with representative quotes to highlight the students’ experiences.

**Table 2:** Overview of subcategories and categories

Subcategories	Categories	Theme
Perceived core message, Negative attitudes, Positive attitudes	Conceptual understanding and attitudes towards PD	When theory meets reality, a mismatch in communication
Perceived problems with scenarios and communication exercises, Positive experiences with communication exercises, Mentorship during exercises, Reflections on feedback	Experiences from theoretical communication training	
Impact of role models, General challenges with application, Experiences when using the model	Reality of clinical transition	
Preclinical PD education, Communication training, Internship	Wishes and perceived needs	

### Category 1: conceptual understanding and attitudes towards PD

*Perceived core message* of the PD course according to medical student’s opinions diverged. Students talked about patient centered approach being at the core what PD course was trying to convey.

*” Well, to see the patient more as an individual and not just as kind of… the illness or the condition that they have sought medical help for, but to understand that there is a human there, who has thoughts and feelings and concerns and stuff like that, this must be kept in mind during the consultation” [No. 9]*.

Self-reflection and expectations on how to communicate were also identified as a part of the core message of the course.

*” Um, that is a hard question but what seems to be pervading is that you’re supposed to be aware of the position you have as a future physician” [No. 12]*.

The participants’ attitude towards PD and communication skills education varied. Some students expressed that they initially had a negative attitude towards the PD course, but it improved as the course progressed. Changing of attitude reflected the shift in perspective due to clinical connection and focus on communication exercises that the students deemed valuable.

*Negative attitudes* were expressed as struggling to see the usefulness of the course when the connection between theory and clinical reality was obscure during pre-clinical semesters with no patient contact, and that the exercises were perceived as too simplistic to feel valuable. Students expressed that the lectures during PD course sometimes felt pseudo-scientific and vague when contrasted with other pre-clinical courses.

*” Well, it’s because during the earlier semesters, there is so much theoretical knowledge that you are supposed to learn, and you don’t have that much contact with patients really. In that stage it might be somewhat difficult to understand why you are supposed to have the PD course and why it’s useful.” [No. 9]Positive attitudes* revolved around general positivity towards the course and in particular the self-reflection aspect of the education.

*” Yeah well, I still think that my attitude has become much better the further we proceed, since the course become less abstract as well.” [No. 15]*.

*”…as you meet more patients, you understand more and more about the purpose of PD, what use you have for it.” [No. 9]*.

### Category 2: experiences from theoretical communication training

The medical students describe a variety of experiences both positive and negative, spanning from the structure and exercises included in the advanced communication skills during their 4th year to mentorship and feedback from the course tutors.

*Perceived problems with scenarios and communication exercises* included feelings of being judged during consultation exercises in groups, when fellow students and tutors gave their feedback after roleplay. Students described difficulties with roleplaying due to lack of medical knowledge, which pushed them to shift from focusing on learning to performing.

*” I get so self-conscious when everyone in the room is looking at me and now I’m going to follow a template and it becomes kind of so focused on “now I’m practicing”. So that it doesn’t become natural, that’s what I think is difficult.” [No. 6]*.

Furthermore, the participants experienced that the scenarios only reflected the clinical reality in primary care and that reflection exercises were too repetitive, leading to students losing focus.

*” Um…maybe because the exercises we had were very focused on primary care and there were very open dilemmas and very diffuse problems, that it can be very complex situations that are described.” [No. 3]*.

*Positive experiences with communication exercises* revolved around perceived benefits of practicing consultations and use of the PCCM preparing the students for meeting patients during forthcoming internships. The students expressed that they felt less pressure to perform and could focus more on learning when in an educational environment, in contrast to interactions with real patients. After the course in communication skills, they felt better prepared to face more complex patients. The opportunity to self-reflect and reflect on exercises where fellow students participated were emphasized as valuable. When students contrasted advanced communication skills course with PD courses during pre-clinical years, they were positively surprised finding the communication exercises more useful.

*” Well, what I appreciate most about the communication skills course is the opportunity we get, to practice consultations and especially when you might not have done it before, it’s good and I think you have much use for it, and kind of reflect about what I did that was good or bad.” [No. 15]*

*Mentorship during exercises* was highly valued by students, especially hearing about older colleagues’ experiences and how they would handle certain situations and patients. This was contrasted to trying things out without much guidance beforehand, which was compared to being blindfolded.

*” I had a teacher that had worked a lot in primary care, and he gave very wise comments, kind of explained how he usually would do it. I thought it was very important to listen to that. He knows what works and what kind of makes it as easy as possible if it’s bad news that must be given. I thought it was very educational.” [No. 10]*.

During *reflections on feedback* the participants expressed that feedback received during communication training was not always perceived as valuable. The students couldn’t relate to the feedback in some cases, struggling to see usefulness of generic feedback without specific points or with focus on insignificant details. In contrast to that, there were students who felt that the feedback they got was valuable and helpful.

*” Then it depends on how your teacher is, if he/she remarks on details or looks at the whole, then it’s different.” [No. 2]*.

### Category 3: reality of clinical transition

When going from a strictly educational environment when practicing the PCCM and roleplaying with fellow medical students to the clinical reality at an internship with patients, the students conveyed that a number of different factors influence their application of communication skills.

A major point was the *impact of role models* affecting if students felt able to use the PCC model. Some students expressed that they felt like they wasted their clinical tutors’ time when applying the PCCM and were not comfortable using it while their tutors were present. Students perceived that stressed tutors inhibited the use of the PCCM because students did not want to be a bother and take up more of their time than necessary. This led to the exclusion of questions surrounding psychosocial matters since they were perceived as non-essential to the patient visit and the students adopted a consultation style more like shown by their clinical tutors.

*” If a physician is present, that is siting and observing me, then I try to do it as you are supposed to at the ward, so I don’t, well I have never been told off of course but you get it, then I try to keep to their methods. But if I am talking with the patient on my own, I speak totally differently and do it in a completely different way because then I don’t feel any time pressure since I’m a student.” [No. 12]*.

The students also described that stressed tutors could interrupt consultations that were conducted by the student and start asking questions when they felt the consultation was too slow.

*” I know that the majority of my tutors have a shortage of time and might not like some of the things I ask about and kind of don’t think that it’s necessary to ask about then and there, some can even interrupt and take over. It’s very annoying.” [No. 15]*.

Some students perceived their clinical tutors as uninterested in teaching and experiencing that they didn’t provide any mentorship of substance during the internship. The students described a lack of meaningful feedback after their consultations with patients. Opinions regarding mentorship and to what degree students received guidance from older colleagues varied from no or little to satisfactory.

*” When I had out-patient visits with a senior physician, at first, she was very grumpy and not especially glad, and I didn’t get to do that much at all during consultation or examining the patient, she was not that nice. Then afterwards I asked why I didn’t get to do anything, she answered that we students are so indifferent and don’t show any interest. Well, then I spoke up and said that you are the ones who don’t want to teach us anything as it seems. At that time, I had attended out-patient visits for two days and heard from others that they didn’t get to try to do anything on their own either and that they had bad mentoring, so then she changed completely and was very nice and explained everything and I got to do everything.” [No. 13]*.

According to the students, many clinical tutors lacked patient centeredness during their own interactions with patients and were negligent about exploring the patient’s psychosocial aspects. Some students expressed that they had started to forget how to use the PCCM since it was not practiced by surgeons they encountered during the internship.

*” First and foremost, it kind of feels like, those that have been a physician for a while do it very sloppily. Because they know exactly what questions they want answers to and then they might ignore other information that might be important. They are really fast and maybe just push through.” [No. 2]*.

*General challenges with application* were the hinders which the students perceived when considering application of the PCCM. Too few physician mentors and general lack of structure during surgical internship was the reasons for less time with patients and fewer opportunities for the students to take responsibility for their own patients. The described experience of no or limited patient interactions at the internship was frequent; in contrast, some students felt they had a satisfactory number of interactions with patients. Medical knowledge and lack of thereof affected consultations. Students did not feel comfortable with the physician’s part of the consultation, which involves asking questions about symptoms and past medical history, and therefore choose not to take on as many patients as they could have otherwise. Feeling unaccustomed to the PCCM and being nervous applying it was considered a barrier to using the model.

*” It’s very unstructured at the surgical internship in general. So, I don’t know if the surgeons are aware that we are going to be there, which is really confusing because there are always students at a university hospital.” [No. 11]*.

*Experiences when using the model* were generally positive. The participants saw the consultation model as a foundation to fall back on and a good structure which helps them avoid missing important information during history taking.

*” If you use the model as a foundation, you can always fall back on it, no matter how difficult, upset, sad or angry the patient is.” [No. 9] *There were also negative opinions centered around how focus on PCCM model during consultations led to interrupted flow and that the interaction could feel forced. When contrasting receipts and pseudo-receipts (repetitive phrases, sounds or body language which according to the PCCM do not signal attentiveness), students experienced frustration as they considered pseudo-receipts a normal part of communication in general. Some students perceived talkative patients as difficult because they felt it was hard to avoid being overwhelmed by such patients while asking open and explorative questions during the initial part of consultation.

*” There are things I don’t really know how they have reached conclusions about, like giving pseudo-receipts, like saying okey. You do it with everyone you talk to, no matter who it is, it just feels like, how can it be wrong? I don’t really understand how they have reached that conclusion, the model just says, don’t say this.” [No. 4]*.

When outlining their experience applying the model with real patients, some students encountered patients that did not respond well to the PCCM. The initial open question regarding why they attended the physicians-appointment was sometimes met with skepticism and questioning regarding if the student wasn’t well prepared for the visit. Furthermore, some patients responded negatively towards questions regarding ideas, concerns, and expectations. Students described situations where they perceived that the patient got worried after beingasked about concerns.

*” And especially when you have time, I feel like it doesn’t always work, making the patient’s part so long. Even if I try, most of [patients], some anyway, do get annoyed. As an example, “could you tell me why you have come here today?” and then the patient responds with “don’t you know, can’t you check, haven’t you read up in my notes beforehand?” [No. 10]*.

The application of the model in situations other than in primary care, or as the students describe it, during explorative or investigative consultations was described as difficult. Students felt that PCCM wasn’t applicable to situations encountered at the surgical internship, for example ward rounds.

*” Partly I think that their models and the way you are supposed to go about it when you have the patient’s part and then the physician’s part and the shared part, it is more applicable in primary care, where you have more time, that’s where I think it works great.” [No. 10]*.

### Category 4: wishes and perceived needs

Students argued that the communication exercises and the internship needed to change to help them effectively with the clinical transition of communication skills.

Students described that *preclinical PD education* needed to reach those students that needed this course the most, rather than focusing on students who already embraced the patient centered approach and are actively engaged in the course. Some wished for preclinical PD course to be taught during later years to obtain a clearer clinical connection.

*” I think that they aim [the course] towards those that don’t really think about the things taught during preclinical PD, and they kind of miss the mark and instead end up preaching to the choir.” [No. 11]*.

*Communication training* could according to the students be improved by including scenarios that the students are likely to face at the surgical internship. The participants expressed that they wished for scenarios with a greater variety of contexts and patients, as well as more input from tutors regarding how they would have handled the situation themselves. Smaller groups during exercises were also requested, as this would lead to more hands-on practice and that they would feel more comfortable and less judged.

*” I believe practicing ward rounds with role playing would be beneficial. But then the scenarios must reflect reality in a way that really conveys how it is on the ward at the hospital, so we are not talking about ideal rounds’ scenarios in a dreamworld because you might not have a use for that kind of role play, but it should be as authentic as possible” [participant No. 9]*.

Needs related to surgical *internship* highlighted expectations of more feedback from clinical tutors after consultation. The students argued that it would be beneficial if tutors were accustomed to using the PCCM and able to provide feedback on its application by students. A recurrent wish was for more opportunities to practice the consultation with patients with the PCCM; i.e. more opportunities for students to be responsible for their own patients under supervision, rather than observing interactions of their tutors.

*” I think it might be beneficial with support and feedback in the beginning, when you start standing on your own feet and testing things out, perhaps just more feedback and be able to meet a lot of patients and learn how you want to approach it.” [participant No. 14]*.

## Discussion

The main finding of the study, as the theme suggests; When theory meets reality- a mismatch in communication, shows that communication skills training and the students preconceptual understanding of consultation practice collide with experience from clinical internship, influencing the transfer of skills negatively. The theme identified in this study includes four categories with linear and temporary relationship, with early education in professional development and communication skills during pre-clinical years affecting perception of advanced communication training during year 4, which in turn collides with the reality of clinical transition to the surgical ward, with students’ reflection on needs and wishes after the process.

To understand how students perceive the larger framework surrounding the course in communication skills, we have asked how they understand the core message of the course in professional development spanning the entire medical program. The way the students conceptualize the core message shows a divergence in understanding. Some students understood PD as a training in communication skills, while others included a patient centered approach, self-reflection, and leadership. The results raise a question if failing to convey the content and learning objectives of the preclinical PD course could affect how students embrace the patient centered approach during later stages of their education [16]. However, while identifying clear objectives is generally recommended in higher education, there is weak support in literature that learning objectives and their use do affect student performance [17].

Our findings indicate that the preclinical part of the PD course was perceived as superfluous, irrelevant, or pseudo-scientific by some students. The course was perceived as having wrong approach, tailored to students who already valued the content of the course and were likely to achieve its objectives while failing to engage reluctant students. While both findings correlate with the heavy emphasis on the biomedical viewpoint, and a down prioritizing of the psychosocial aspects in line with Hvidt’s findings [18], seeing the content as pseudo-scientific could impact the student’s adoption of the patient centered approach. Tailoring the course to each participant unique strengths and weaknesses is not feasible but acknowledging and challenging the students’ preconceptual views and emphasizing benefits of PCC for the patient supported by research [2] could potentiality improve the attitudes and affect learning outcomes.

Overall, these results suggest that the students did not find the preclinical PD course containing introduction to patient-centered care meaningful. When examining this finding through the lens of transformative learning theory, the outcome of the course would ideally be affecting students’ identity through incorporation of PCC as part of self. Integral to transformative learning in healthcare is acceptance that the students are going to experience degrees of vulnerability during the learning process, which will induce both positive and negatives attitudes towards the training [19]. While it is important to strive to improve students’ motivation, the preclinical PD course could be viewed through this lens as an initial stage in their development, where students start navigating the ‘disorienting dilemmas’ and create their own meaning.

The students expressed difficulties with prioritizing learning and ended up focusing on performance during roleplay exercises. Performance according to participants meant delivering the right medical knowledge, focusing on identifying correct diagnosis and prescribing appropriate treatment. The group size of nine students was seen as too big and a hinder to working in a tightly knit, congenial environment focused on learning. The medical students’ proclivity for perfectionism and associated psychological distress, while not mentioned by participants, could be one of the factors enhancing their feelings of being judged during roleplay [20]. When conducting group exercises the importance of creating an accepting and encouraging environment cannot be understated, since group learning is associated with ingroup comparison and performance stress, especially in highly performance focused environments such as medical school [21, 22].

The fact that roleplay scenarios mostly reflected primary care visits was a recurrent critique. Students expressed that the exercises did not reflect the situations they would face in the surgical internship. This could be interpreted as an inability to adapt newly learned skills to situations other than explicitly practiced, which in turn would imply that the students haven’t reached high enough familiarity with the model to transfer the theoretical knowledge into practice [23]. It could also reflect a too rigid educational environment, where the adaptability of the model isn’t emphasized and strict adherence to the model is promoted instead.

The participants described several factors that affected them when starting to apply the PCCM in clinical settings. Tutors impact on the transition of communication skills was highlighted as a major point, in keeping with how learners acquire skills, attitudes and identity when participating in communities of practice [5]. Students perceived their tutors as stressed, which affected their use of the PCCM, since they did not want to be a bother by taking more time by exploring psychosocial matters with the patients. This correlates with the students experience of clinical tutors lacking patient centeredness and rarely exploring psychosocial aspects themselves. In addition to that, students described having consultations interrupted by tutors and being forced to move directly to the physician’s part of the consultation, with direct questions about symptoms, omitting patient’s agenda and signaling that use of the consultation model and patient centeredness lack relative importance and is not prioritized. This correlates further with studies of variables that affect transfer of skills in other contexts, in particular the need for social support when applying skills in the work environment [24–26]. The importance of the transfer climate affects the success of the transfer directly, with climate acting as a moderator between the organization where the skills are applied and the individual applying it and affecting the motivation of the individual i.e., intention to implement [27].

While the students’ thoughts on the reasons behind the lack of patient centeredness of their teachers were not the scope of this study, the participants identified stress and pressure of clinical workload as one of the barriers. In recent systematic review on shared decision making (SDM), which falls under the umbrella of PCC, lack of formal training and professional role and identity were identified as factors blocking implementation. Physicians who see themselves as educators or collaborators were more likely to adopt SDM in their practice. Other factors were beliefs about capabilities of patients and environmental and contextual aspects, such as lack of time or noisy and busy surroundings [28]. In the systematic review on communication skills of surgeons, the surgeons were found to provide satisfactory information about surgical conditions and treatments for their patients, while at the same time lacked the necessary skills to explore their emotions or concerns [29]. Recently, several studies describing introduction of PCC courses to surgical residencies have been published [30–33], but globally, the inclusion of communication skills in the surgical curriculum is still sporadic, contributing to cognitive dissonance of students who meet unprepared trainers [34, 35].

On the other hand, most students described a positive experience using the PCCM after first attempt to practice the model with real patients. They described the PCCM as a useful tool, which gave structure to their consultations, but at the same time they felt it was unnatural to communicate following the model in a strict way. However, students experienced that they were offered too few opportunities to speak to patients to be able to practice PCC independently, which affected the transition negatively. Another internal barrier to implementation described by students was being uncomfortable while using the model if they didn’t have enough medical knowledge to handle the physician’s part of the consultation, and therefore opted out of opportunities to consult own patients. Limited opportunities to apply newly learned skills have been highlighted as the biggest hinder to implementation or transfer of skills [25, 36]. ‘Learning by doing’ is a prerequisite for the students, who need to practice PCC with patients if effective transfer of communication skills is to take place.

When the students described their perceived needs and wishes, they mainly focused on two areas that needed improvement.

Changes to the communication exercises that the students wished for was smaller groups, more variety in scenarios and more input from tutors. While changing the group size would effectively mean doubling of teacher resources, scenarios incorporating less primary care based, explorative consultations and mirroring situations and challenges which might be encountered during the ward rounds could be added easily. Alternatively, emphasis on learning how the PCC can be adapted to different situations during the course might achieve the same result.

The students expressed a need for tutors on the surgical internship to be familiar with the PCC model and wished for more feedback after consultations with patients. More opportunities to consult patients of their own were also expected. It seems obvious that to be able to teach something you need to know it first. As Chiaburu et al. mentions [37], the role of supervisors regarding transition of skills is one of the most influential factors. If the students were exposed to the PCC model to a larger extent i.e., when observing their clinical tutors communicating in a patient centered way, and getting relevant feedback with focus on the model, this would arguably enhance the transfer of communications skills.

### Strengths and limitations

The findings of this study provide contextual knowledge about medical students’ transfer of communication skills at a university hospital in Sweden. Although contextual, the findings could be transferred to other educational settings with similar setup with communication skills training followed by hands-on practical internships.

One of the possible limitations of the study was the fact that the interviewer was a medical student. This could affect the process with biases due to own lived experiences in the same context. On the other hand, shared experience was a reason for greater access and trust by participants. Recruitment of study participants was done on an opt-in basis, although we have managed to capture both positive and negative viewpoints.

We recommend that future research on the subject focuses on observational studies, to further anchor the student’s experiences in their proper context.

## Conclusions

Preparing the students through theoretical and practical training should reflect the reality they will face when entering clinical practice. When educating medical students as a group, their proclivity for perfectionism, high performance environment and achievement-related stress should be taken into consideration. The role of tutors being role models, offering guidance, giving feedback and support plays a major part in facilitating transfer of communication skills. To enable transfer to a larger extent, the environment needs to promote patient centeredness, as well as students need more opportunities to practice communication with their patients.

## Data Availability

The dataset generated and analyzed during the current study are not publicly available considering the integrity of the participants but are available from the corresponding author on reasonable request.

## Abbreviations

PCC: Patient centered communication
PCCM: Patient centered communication model
PD: Professional development
